# Training the Blind

**Published:** 1920-09-25

**Authors:** 


### THE PUBLIC WELL-BEING.

### Training the Blind.

### NEW GOVERNMENT OPPORTUNITIES.

In our news columns recently we published a summary of the second report of the Advisory Committee on the Welfare of the Blind, issued by the Ministry of Health. We have now before us an interesting memorandum which the Board of Education have sent out containing and explaining regulations in the training of the blind. The Board point out that the many problems connected with the welfare of the blind have recently received much careful consideration. Acting on the recommendations contained in the report of the Departmental Committee on the Welfare of the Blind, the Local Government Board appointed in 1918 an Advisory Committee to advise them in all matters affecting the welfare of the blind. In August last year the Ministry of Health issued regulations under which grants are payable in aid of various activities designed to promote the welfare of the blind, and notably in aid of schemes for assisting the blind worker to pursue a trade either in a special workshop or in his own home. If, however, the blind worker is to be able to take any adequate advantage of the facilities thus afforded, it is essential that he should first undergo a thorough course of training. A blind child on leaving school at sixteen is not an efficient worker, and cannot be expected to be a wage-earner. Not only must the pupil receive a thorough training in all branches of the industry he is to pursue, but a considerable period is necessary to enable him to acquire the speed essential to a successful worker.

#### An Essential Link.

The training institution is in fact now recognised by the Board as "an essential link between the special school and the workshop." They point out in this connection that while local education authorities have largely fulfilled their duties in regard to the provision of suitable education for the blind child, they have not fully appreciated the need for his further training after he leaves the special school. Account has also to be taken of the needs of blind persons who, owing to the age at which they became blind or other circumstances, have not attended a special school, but who are capable of benefiting by attendance at a course of training. No doubt the development of adequate facilities for training has been somewhat hindered hitherto by the lack of suitable opportunities for the blind worker to follow the trade which he has learned.

#### Removing a Handicap.

It may be anticipated, however, that the action now being taken by the Ministry of Health will go far to remove this difficulty, and local education authorities and managers of institutions should accordingly seriously consider what provision is necessary to secure that the blind worker is not handicapped in life through lack of opportunities for adequate and efficient training. The increased provision of special schools for epileptic and cripple children which will result from the passing of the Education Act, 1918, will also lend added importance to the provision of suitable facilities for the instruction and training of these particular groups of students.

#### Probable Openings.

In determining the trades for which instruction and training can best be provided, careful consideration must be given to probable openings for employment, either in a profession or in the open markets of industry and commerce, locally or otherwise, or in special workshops, or in home industries. Under whatever conditions the students may be likely subsequently to be employed, it is necessary that their training should not be confined to gaining facility in manipulation. Opportunity should be afforded for instruction in the commercial aspects of the trade, the source and cost of materials, their preparation for use, buying and selling, and generally the method of conducting the trade. The course of instruction should also provide for a continuance of the student's instruction in general subjects and for appropriate physical training.

#### Length of the Course.

The length of the course will necessarily vary according to the nature of the trade for which preparation is afforded. Admission should be confined to students who are prepared to stay for the whole course and are likely to be able to do so with advantage. Students who have not previously attended a special school may be admitted to an approved course only if the Board are satisfied that their age and attainments are such as to make it likely that they will profit by attendance at the course. A student should not be allowed to continue the course if he has ceased to make progress; on the other hand, premature transfer to the workshop should not be allowed. The Board have increased substantially the grants payable in aid of the training of students of these special types, and it has been decided to continue to pay grants direct to an institution not provided by an authority in respect of students for whom no fee has been paid by an authority. The new regulations, in the first place, provide for the payment to a local education authority of a grant of one-half of their net expenditure in (*a*) approved courses in recognised institutions provided by the authority, and (*b*) payment of fees of students attending approved courses at a recognised institution not so provided.

